# Metabolism in Cancer Stem Cells: Targets for Clinical Treatment

**DOI:** 10.3390/cells11233790

**Published:** 2022-11-26

**Authors:** Gui-Min Wen, Xiao-Yan Xu, Pu Xia

**Affiliations:** 1 Department of Basic Nursing, College of Nursing, Jinzhou Medical University, Jinzhou 121001, China; 2 College of Basic Medical Science, China Medical University, Shenyang 110122, China; 3 Biological Anthropology Institute, College of Basic Medical Science, Jinzhou Medical University, Jinzhou 121001, China

**Keywords:** metabolism, cancer stem cell, ALDH1, ferroptosis, tumor microenvironment

## Abstract

Cancer stem cells (CSCs) have high tumorigenicity, high metastasis and high resistance to treatment. They are the key factors for the growth, metastasis and drug resistance of malignant tumors, and are also the important reason for the occurrence and recurrence of tumors. Metabolic reprogramming refers to the metabolic changes that occur when tumor cells provide sufficient energy and nutrients for themselves. Metabolic reprogramming plays an important role in regulating the growth and activity of cancer cells and cancer stem cells. In addition, the immune cells or stromal cells in the tumor microenvironment (TME) will change due to the metabolic reprogramming of cancer cells. Summarizing the characteristics and molecular mechanisms of metabolic reprogramming of cancer stem cells will provide new ideas for the comprehensive treatment of malignant tumors. In this review, we summarized the changes of the main metabolic pathways in cancer cells and cancer stem cells.

## 1. Introduction

In the past 30 to 40 years, a small unique population of cancer cells with tumor-initiating and self-renewing abilities was named as cancer stem cells (CSCs) [1]. Scientists have been able to find heterogeneous cells in different tumors that have cancer stem cell characteristics [2]. Cancer stem cells have the ability of self-renewal, unlimited proliferation and multipotent differentiation, and play an important role in cancer survival, proliferation, metastasis and recurrence [1,2]. The therapeutic resistance of CSC is one of the important reasons for the failure of tumor treatment. The therapies targeting CSC are expected to solve the clinical bottleneck of tumor recurrence and metastasis [1,2]. More than 40 established CSC markers have been used separately or jointly for sorting cancer stem cells [3]. However, until now, we have not known which subgroup are cancer stem cells. In fact, the heterogeneous cells we found in our studies can only be called cancer stem-cell-like cells. In our opinion, cancer stem cells cannot be defined accurately depending on surface markers or functional markers. In order to define cancer stem cells, four elements of evidence of identification need to be considered. The first piece of evidence is widely used markers, such as CD133, CD44 and ALDH1 [4]. The second piece of evidence is the gene differences between cancer stem cells and cancer cells [5]. The third piece of evidence is the abnormal metabolism state of cancer stem cells [6]. The fourth piece of evidence is the effects of cancer stem cells on the cells in the tumor microenvironment (TME), especially immune cells [7]. The metabolism of cancer stem cells has been a research hotspot in recent years. More and more studies have shown close relationships between metabolic reprogramming and stemness [8,9,10]. In this review, we will summarize the metabolic phenotype of cancer stem cells and further present potential therapeutic targets for treatment.

## 2. Overview of Cancer Metabolism

**Figure 1:**
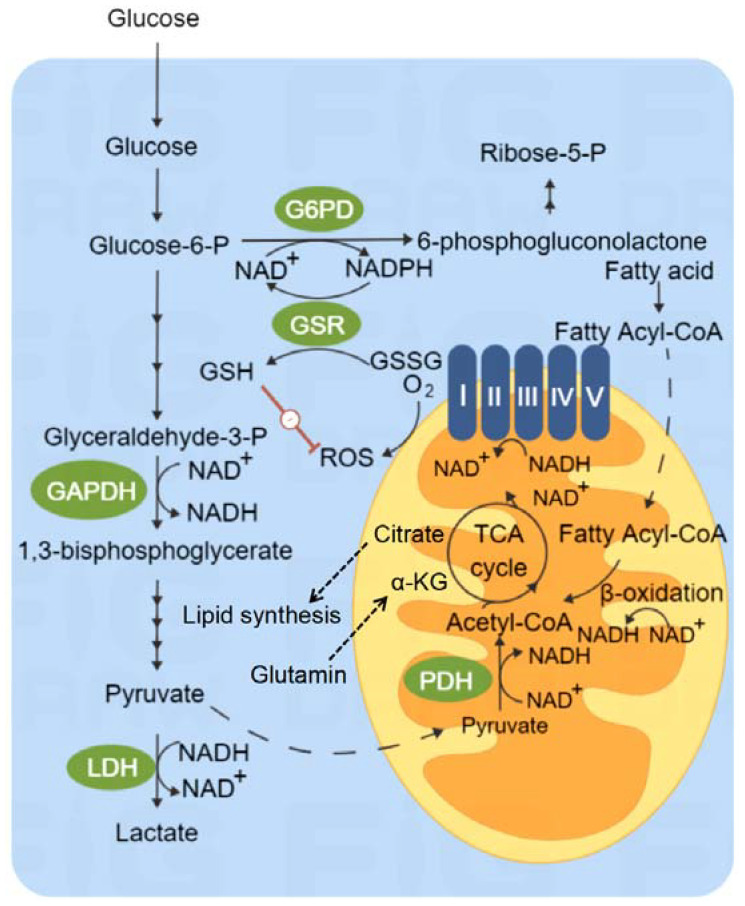
Metabolic pathways in cells. Glucose is broken down into pyruvate and ATP in cells, which is called glycolysis. Nicotinamide adenine dinucleotide (NAD^+^) from glycolysis will maintain the levels of reduced glutathione (GSH). GSH can reduce the levels of reactive oxygen species (ROS) and protect cells from the oxidative damage caused by free radicals. The tricarboxylic acid (TCA) cycle is a necessary pathway for the complete oxidative decomposition of glucose, fatty acids and amino acids. Some intermediate metabolite molecules formed by the TCA cycle are also precursors for the synthesis of many important biological molecules. Fatty acids are synthesized from acetyl CoA produced from TCA cycle. NADH: nicotinamide adenine dinucleotide reduced form; NAD: nicotinamide adenine dinucleotide; GAPDH: glyceraldehyde-3-phosphate dehydrogenase; LDH: lactate dehydrogenase; PDH: pyruvate dehydrogenase; ROS: reactive oxygen species; GSH: reduced glutathione; GSSG: oxidized glutathione; TCA cycle: Tricarboxylic acid cycle.

Metabolic reprogramming refers to the change in the metabolic mode of cancer cells compared with normal cells [11]. The reprogramming provides sufficient energy and nutrients for cancer cells and cancer stem cells [11]. The high proliferation and metastasis of cancer cells are signs of metabolic disorders [12]. Otto Warburg reported on the increased utilization of glycolysis in cancer cells to meet energy requirements in 1924 [13]. In normal cells, glucose mainly generates pyruvate through glycolysis [14]. Pyruvate is transported into mitochondria and generates adenosine triphosphate (ATP) through tricarboxylic acid (TCA) cycle-coupled mitochondrial oxidative phosphorylation (OXPHOS) to supply energy for cancer cells [14]. Warburg found that the growth of normal cells mainly depends on the TCA cycle in mitochondria to convert glucose into energy ATP, while cancer cells have a different metabolic phenotype from normal cells; that is, cancer cells prefer anaerobic glycolysis even in the presence of oxygen [13]. Since the Warburg effect was discovered, abnormal metabolism has been a never-ending topic in the field of cancer research. At present, researchers not only pay attention to glucose metabolism, but also pay attention to other metabolic types, such as glutamine metabolism (Figure 1) [15]. Abnormal metabolism usually produces a higher expression of hypoxia-inducible factor-1 (HIF-1) that is a critical premise for epithelial to mesenchymal transition (EMT) [16]. The metabolism-EMT-metastasis network has been delineated in recent studies [17,18,19]. EMT refers to the process of epithelial cells changing into mesenchymal cells under physiological or pathological conditions, which endows the cells with stemness characteristics, including metastasis, differentiation and proliferation. It not only plays a key role in the development process, but also participates in the process of tissue healing, organ fibrosis and cancer occurrence [20]. In the following sections, we will summarize various metabolic abnormalities of cancer stem cells.

## 3. Glucose Metabolism

Glucose metabolism is the first and most widely studied metabolic abnormality in cancer cells (Figure 1) [21,22,23,24]. Normal cells metabolize glucose into carbon dioxide and water through oxygen-consuming mitochondrial metabolism in physiological conditions, but convert glucose into lactic acid (glycolysis) in a low oxygen and anaerobic environment [25]. In all conditions, glycolysis is the priority selection for cancer cells to generate energy, termed the “Warburg effect” [26]. Compared with cancer cells, CSCs have higher rates of glycolysis [27]. Glucose induces the expression of key genes and the activity of glycolytic enzymes in the glucose metabolism pathway, such as hexokinase 1 (HK-1), HK-2 and glucose transporter 1 (GLUT1) [28,29,30]. High oxygen and low glucose decline the CSC population from breast cancer, ovarian cancer, lung cancer and colon cancer cells [31,32,33,34]. Breast cancer stem-cell-like cells have higher activities of pyruvate kinase M2 (PKM2), lactate dehydrogenase (LDH) and reactive oxygen species (ROS) than other breast cancer cells [35]. ROS is a key factor in maintaining the stemness of CD44^+^ breast cancer cells through upregulating the expression of CD44 and signal transducer and activator of transcription 3 (STAT3) [36]. Hepatocellular carcinoma and nasopharyngeal carcinoma stem cells show a preference for glycolysis during radiotherapy [37,38]. Based on the evidence above, CSCs usually show an increase in glucose uptake and lactate production, together with a decrease in mitochondrial respiration. However, there seems to exist differences regarding the metabolic state in heterogeneous tumors. Compared with glioma cells, glioma stem cells consume less glucose and produce less lactic acid, while ATP production is very high [39]. Low glycolysis levels and high oxidative phosphorylation in glioma stem cells are due to abundant mitochondria [39]. These results further proved that the glycolysis pathway can be used as a target for anticancer therapy.

## 4. Mitochondrial Metabolism

Mitochondria are key organelles to generate ATP and maintain cell metabolism [40]. Although other processes, such as glycolysis, can generate ATP in normal cells, more than 90% of ATP is generated in the mitochondria through oxidative phosphorylation (OXPHOS) pathway (Figure 1) [41]. OXPHOS is suppressed in most CSCs for losing the function of mitochondria [42]. The upregulated OXPHOS pathway is closely related to the process of EMT and tumor metastasis [43]. In a relatively nutrient deficient environment, the oxidative stress reaction promotes tumor cells to produce lactic acid and other metabolites through glycolysis, and further supply the anabolism of tumor cells with OXPHOS as the main metabolic pathway, to promote tumor growth [44].

Dichloroacetate (DCA) has been used to treat children born with mitochondrial diseases since the 1960s [45]. It can inhibit pyruvate dehydrogenase kinase (PDK)-induced phosphorylation of pyruvate dehydrogenase (PDH), promote the conversion of pyruvate to Acetoacetyl coenzyme A (Acetoacetyl-CoA, Acetyl-CoA) and change mitochondrial metabolism from glycolysis to oxidative phosphorylation, so as to reduce cell proliferation, promote apoptosis and inhibit glioblastoma growth [46]. DCA increases mitochondrial depolarization and ROS production, and induces apoptosis in CD133 positive glioblastoma stem cells both in vitro and in vivo [47]. In addition to ATP, mitochondria are also the main place to produce ROS [48]. ROS is a highly reactive metabolic molecule, including superoxide radical anion, hydrogen peroxide and hydroxyl radical [48]. ROS can be used as the second messenger of many stimulating factors to participate in the regulation of cancer stem cell migration, proliferation, stemness maintenance and differentiation [49]. In physiological status, the production and clearance of ROS are in dynamic equilibrium with the action of the antioxidant system [48]. Excessive ROS accumulation causes oxidative stress, DNA damage and tumorigenesis [48]. Compared with breast cancer cells, breast cancer stem cells (BCSCs) have a high ability to clear ROS and keep ROS at a relatively low level for the high expression levels of superoxide dismutase, catalase, and glutathione peroxidase [50]. Based on this finding, ROS regulation in CSCs may be a potential anti-tumor treatment for killing CSCs and eliminate tumors.

## 5. Glutamine Metabolism

Glutamine is an important non-essential amino acid required for cell proliferation [51]. It is not only the substrate for the synthesis of hexosamine and nucleic acid, but also can enter the oxidative phosphorylation pathway to produce energy through TCA cycle under hypoxia, which is also an important metabolic mode necessary for tumor cells to maintain their own activity (Figure 1) [51]. Glutamine can be converted into glutamate under the action of glutaminase for the synthesis of fatty acids and reduced glutathione (GSH) [52]. The antioxidant glutathione can maintain the balance of redox reaction in cancer cells and help the cells resist oxidative stress [53]. The low ROS level of CD44-positive gastrointestinal CSCs may be related to antioxidant-reduced glutathione [54]. CD44 interacts with and stabilizes the glutamate cystine transporter, xCT (SLC7A11), and promotes the synthesis of GSH in gastrointestinal CSCs, thus improving the cells against ROS [55]. As a functional group of glutamate/cystine reverse transporter, xCT transports extracellular cystine into cells [56]. To maintain intracellular glutamine levels, tumor cells overexpress xCT to meet the needs of rapid proliferation [57,58,59]. It has been confirmed that the expression level of xCT is increased in various types of tumors, such as hepatocellular carcinoma [57], colorectal cancer [58] and breast cancer [59]. The high expression of xCT is associated with the late progression of lung cancer and the reduction in the 5-year survival rate of patients [60]. Downregulation of xCT can weaken the proliferation and invasion ability of non-small cell lung cancer cells in vivo and in vitro [60]. xCT also promotes the invasion and proliferation of prostate cancer cells [61]. xCT overexpression changes the morphology and cytoskeleton of glioma cells, accompanied by high migration, invasion and cell adhesion [62]. xCT also upregulates the stem cell markers’ expression, such as Nanog, Musashi-1, Sox-2 and nestin in glioma cells [62]. xCT expression promotes GSH synthesis and indirectly activates Nanog to enrich CSCs in breast cancer [63]. The above results show that xCT can induce the expression of CSC markers and promote tumorigenesis. It means xCT is a candidate gene for small molecule-targeted drugs.

## 6. Lipid Metabolism

Fatty acid (FA) metabolism is also one of the important metabolic pathways required by tumor cells to maintain their growth activity (Figure 1) [64]. In aerobic conditions, fatty acids will be oxidized and decomposed to release energy [65]. This process is called fatty acid oxidation (FAO) [65]. Carnitine palmitoyltransferase 1α (CTP1α) is the key rate-limiting enzyme of FAO, which catalyzes the transfer of long-chain acyl groups from coenzyme A to carnitine and transports long-chain fatty acids to the mitochondrial matrix [66]. Fatty acid synthesis uses Acetyl-CoA, a product of glycolysis, the TCA cycle and amino acid decomposition, to synthesize the intermediate product of sixteen carbon and then process it into various fatty acids [67]. The level of unsaturated fatty acids in CSCs is higher than other cancer cells, which inhibits the activities of stearoyl-COA desaturase 1 (SCD1) and acetaldehyde dehydrogenase 1A1 (ALDH1A1) in CSCs, reduces the stemness of CSCs and delays tumor formation [68]. The key lipases of FA synthesis in CSCs, such as ATP citrate lyase (acly), acetyl CoA carboxylase (ACC) and fatty acid synthase (FASN), are increased [69]. These lipases, which regulated by lipogenic transcription factor (SREBP1c), have gradually become ideal markers for CSCs [70]. Expression of acly, ACC, FASN and fatty acid transporter (CD36) are increased, and SREBP1c is also highly activated in liver CSCs [70]. In addition, the analysis of AMP protein kinase (AMPK) related to metabolism showed that the phosphorylation level and content of AMPK in CSCs decreased [71]. This change enhanced the activity of lipase in CSCs and increased the content of malonyl CoA, the precursor of FA synthesis, to improve the synthesis of FA and enhance the metabolism of mitochondria β-oxidation [71]. Glycolipid metabolism in CSCs is connected by acetyl coenzyme A (AcCoA) produced after the oxidation of pyruvate, which can be used to synthesize FA [72]. This connection promotes the self-renewal of CSCs and is a main factor in maintaining the stemness [72].

The mevalonate pathway is an important metabolic pathway of steroid hormones, cholesterol and nonsterol isoprene [73]. This pathway mainly maintains the homeostasis of CSCs’ microenvironment through protein prenylation [74]. HMG-CoA is the rate-limiting enzyme of mevalonate pathway and also is the molecular target of statins, the cholesterol-lowering drugs [75]. Statins interfere with the mevalonate pathway and block protein prenylation, thereby destroying the steady state of CSCs microenvironment and killing CSCs [76]. HMG-CoA is overexpressed in basal-like tumors, and inhibition of the mevalonate pathway by simvastatin can reduce the number of CSCs [77]. The combination of valproic acid and simvastatin can simultaneously regulate the mevalonate pathway and AMPK phosphorylation level to inhibit the Yap oncogene, thereby enhancing the sensitivity of castration-tolerant prostate cancer cells to docetaxel and reducing the drug resistance caused by CSCs [78]. After treatment with metformin, an AMPK activator and a HMG-CoA reductase inhibitor, the number of CSCs in colorectal cancer significantly decreased, while the number of CSCs increased after treatment with mevalonate [79]. The mevalonate pathway can accelerate the proliferation of pancreatic CSCs, while atorvastatin can inhibit this proliferation effect [80]. Medroxyprogesterone acetate (MPA) can increase the expression of CD44 and the activity of ALDH1A1 in BCSCs, enhance the tumorigenesis of CSCs and block cholesterol synthesis, which can reduce the formation of tumor spheres in BCSCs [81].

## 7. Iron Metabolism and Ferroptosis

Due to the increase in proliferation rate and metabolic activity of malignant tumors, tumor cells have a high demand for iron [82]. In order to ensure adequate supply, the absorption of iron in tumor cells increases and the release decreases [83]. The synthesis of transferrin exhibits an autocrine mechanism to support iron supply and tumor cell growth [84]. Overexpression of transferrin receptor 1 (TfR1) has been confirmed in cancer tissues, which is related to the short survival of cancer patients [85]. Inhibition of TfR1 expression significantly blocks tumor growth and metastasis [86]. In contrast, iron transporter expression was reduced in tumor cells and was associated with poor patient survival [87]. Elevated levels of hepcidin are found in different tumor types, inhibiting transferrin (Tf)-mediated iron transport [88]. In addition, iron regulatory protein 2 (IRP2) promotes the proliferation of tumor cells and is associated with the low survival of patients [89]. In various tumors, abnormal iron metabolism was significantly related to CSCs [90,91,92,93,94,95,96,97,98]. Compared with non CSCs, the expression level of transferrin in CSCs of glioma and breast cancer are significantly increased [90,91]. Iron plays a role in the regulation of non-small cell lung cancer CSCs through hydroxyl radicals and promotes tumor invasion [92]. Moreover, iron supplementation can promote the stemness of breast cancer, lung cancer and cholangiocarcinoma cells, while iron chelators inhibit the spheroid formation of lung cancer and cholangiocarcinoma cells [93,94,95]. The results indicated that CSCs need more iron than other cancer cells during tumor formation. Compared with CSCs, cancer cells have low expression levels of TfR and high expression levels of Ferroportin 1 (FPN1) and Hephaestion, resulting in low intracellular iron levels [96]. FPN expression is lower in CD44-positive CSCs than negative cells [96]. CD44 interacts with TfR to increase iron absorption through TF/2Fe^3+^, and FPN downregulation reduces iron outflow, resulting in increased iron levels in CSCs [96]. It indicates that CD44 is not only a surface marker for CSCs, but also can increase the iron in CSCs, and maintain the stemness of CSCs.

Ferroptosis is mainly caused by the abnormal increase in “iron” dependent lipid oxygen free radicals and the imbalance of redox homeostasis [97]. Salinomycin can selectively inhibit tumor growth and significantly reduce the proportion of BCSCs in vitro and in vivo [98]. Ferrimycin, as a salinomycin derivative, has better selectivity than salinomycin, and specifically kills BCSCs by promoting the generation of ROS and inducing ferroptosis [99]. Derivatives of epomycin are highly selective for BCSCs, and inhibit glutathione peroxidases (GPXs) activity and bind with system xc- to block cystine uptake to induce ferroptosis of BCSCs [100]. System xc- promotes GSH synthesis by mediating cystine uptake and glutamate release, thereby protecting cells from oxidative stress damage and maintaining the redox balance of cells [101]. Salinomycin derivatives can induce ferroptosis of BCSCs through targeting lysosomal iron [99]. Ovarian cancer stem cells (OCSCs) are characterized by self-renewal, uptake and retention of excess iron [102]. Alastin, a ferroptosis inducer, successfully reduced the viability of OCSCs by inhibiting the GSH/glutathione peroxidase 4 (GPX4) system, but it had no significant effect on non-OCSCs [102]. Artesunate promoted the generation of ROS in OCSCs in a dose-dependent manner to induce ferroptosis [103]. After the intervention of the ferroptosis-inhibitor, ferrostatin, the death of OCSCs induced by artesunate was significantly reversed [103]. Sulfasalazine, an inhibitor of system xc-, can weaken ROS defense mechanism and thus induce ferroptosis of gastric-cancer stem cells [104]. Salinomycin and docetaxel effectively reduce CD44 expression, promote ROS generation and cause ferroptosis of gastric-cancer stem cells [105]. The CD44v-xCT axis regulates the internal redox reaction of CSCs to treat colon cancer [106]. System xc- deficiency causes cysteine deficiency, inhibits GSH synthesis and thus causes ferroptosis in colon CSCs [106]. Temozolomide in combination with quinacrine (hydroxychloroquine derivative) can induce the death of glioblastoma stem cells through iron dependence and accumulation of lipid peroxides [107]. This combination inhibits tumor proliferation and induced these cells to be more susceptible to chemotherapy and radiotherapy [107]. Sulfasalazine and etoposide can reduce the intracellular GSH level, downregulate GPx4 activity and stimulate lipid peroxidation, leading to ferroptosis in neuroblastoma stem cells [108].

## 8. Metabolism of ALDH1 Positive Cancer Stem Cells

The human ALDH family contains 19 different ALDH-protein subtypes [109]. As important members of the non-P450 oxidase system, ALDH proteins mainly participate in the oxidative metabolism of aldehydes in cells [110]. With nicotinamide adenine dinucleotide phosphate (NADP^+^), it catalyzes aldehydes to generate corresponding carboxylic acids [110]. ALDH1A1, ALDH1A2 and ALDH1A3 are three highly conserved isoenzymes in the ALDH1 family, which can catalyze retinol into retinoic acid and degrade toxic substances to protect cells [111]. ALDH1 is highly expressed in a variety of tumor tissues and leads to poor prognosis of patients, such as lung cancer [112], breast cancer [113], esophageal cancer [114] and colon cancer [115]. ALDH1-positive cancer cells have many characteristics of CSCs, such as self-renewal, multidirectional differentiation, tumorigenicity and metastasis [116]. The drug resistance of ALDH1^+^ CSCs is related to the detoxification of ALDH1 [117]. ALDH1 converts aldehyde phosphoramide, an intermediate metabolite of the oxidative-alkylating agent, cyclophosphamide (CTX), into a non-toxic carboxyl phosphoramide, which plays a detoxifying role on CTX, leading to drug resistance of cancers to CTX [117]. Gastric cancer cells show high ALDH activity and the expression of the notch and sonic hedgehog (SHH) signaling pathway after 5-fluorouracil and cisplatin treatment [118]. ALDH1A1 upregulates the secretion of granulocyte macrophage-stimulating factor (GM-CSF) by activating the TAK1/NF-κB signaling pathway to increase the enrichment of myeloid-derived suppressor cells (MDSCs) in the tumor microenvironment (TME) of breast cancer, and to inhibit the proliferation and activation of killer T cells [119]. ALDH1-positive head and neck squamous cell carcinoma cells inhibited T-cell proliferation more efficiently than ALDH1-negative cells, and inhibited cytokine, interferon γ (IFN-γ), Interleukin-2 (IL-2) and tumor necrosis factor α (TNF-α) [120]. Stemness and drug resistance of ALDH1^+^ cancer stem cells are the key reasons why cancers are difficult to cure, but its molecular mechanism needs further research.

## 9. Tumor Microenvironment and Metabolism

The tumor microenvironment (TME) is composed of tumor cells, stromal cells, fibroblasts, immune cells, extracellular matrix and cytokines [121]. Both cellular and non-cellular factors promote CSCs to participate in tumor recurrence, metastasis and treatment resistance [122]. The TME is very important for cancer stem cells, mainly involving hypoxia, nutrient deficiency, immune cells in the microenvironment and their secreted cytokines, growth factors and hormones [123]. The hypoxia microenvironment can promote the expression of stem cell markers in breast cancer, activate the JAK2/STAT3 signaling pathway and increase the fatty acid oxidation level to maintain its stemness [124]. Hypoxia and glucose deficiency will induce immune cells in the microenvironment to develop into immunosuppressive phenotypes, change the interaction between CSCs and immune cells and thus promote the occurrence and development of tumors [125]. Compared with normal tissues, TME is more acidic, thus triggering angiogenesis and improving the invasion of CSCs [126]. In addition, IL-8 can upregulate the expression of GLUT3, improve glucose uptake and hexosamine biosynthesis and regulate the development of CSCs [127]. Leptin derived from breast adipocytes can maintain the stemness and chemoresistance of BCSCs [128]. TGF-β and cytotoxic T lymphocyte-associated antigen-4 (CTLA4) can help CSCs escape immune monitoring [129].

Tumor-related metabolic reprogramming is not only affected by tumor cells themselves, but also by cancer-associated fibroblasts (CAFs), endothelial cells, mesenchymal stem cells (MSCs), immune cells and their secreted cytokines, growth factors and extracellular vesicles in TME [130]. The energy metabolism process of tumor cells can promote the interaction between CSCs and CAFs [130]. CAFs provide sufficient energy for self-renewal, invasion and metastasis of CSCs [130]. Lactic acid, a tumor metabolite, is mainly discharged from cells through the monocarboxylic acid-transporter family [131]. Cancer cells, CSCs and CAFs express different types of MCTs [132]. CSCs with epithelial cell-like phenotype express MCT-1, while CAFs and other non-CSC tumor cells express MCT-4 [132]. Under hypoxia, CAFs and lactate produced by tumor cells are excreted through MCT-4 and then absorbed by epithelial-like CSCs expressing MCT-1 as substrates for energy metabolism [133]. Endothelial progenitor cells in TME can differentiate into blood vessels and provide oxygen and nutrients for the tumor [134]. In order to generate ATP faster, glycolysis is the preferred pathway for tumor-associated endothelial cells’ formation [135]. Vascular endothelial growth factor (VEGF) mainly supports angiogenesis by activating the glycolytic pathway, while the downregulation of predicted 6-phosphofructo-2-kinase/fructose-2,6-bisphosphatase 3 (PFKFB3) in the glycolytic pathway of endothelial cells inhibits the glycolytic pathway and reduces the efficiency of angiogenesis [136]. In view of the important role of VEGF in angiogenesis, it has become the target for anti-angiogenic therapy.

**Figure 2:**
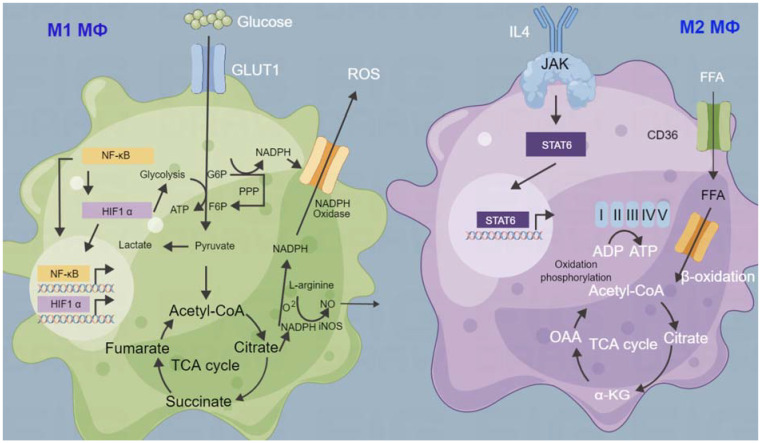
Metabolic difference between tumor-associated macrophage (TAM) M1 and M2 type. Metabolites of cancer cells and stromal cells in the tumor microenvironment (TME) lead to metabolic reprogramming of TAMs and change the composition and function of TAMs. M1-type TAMs have a high level of aerobic glycolytic activity and can produce oxygen species (ROS) to kill cancer cells. M2-type TAMs rely on high levels of oxidative phosphorylation (OXPHOS) to produce cytokines to promote the proliferation of cancer cells. G6P: glucose-6-phosphate; F6P: fructose-6-phosphate; GLUT1: glucose transporter 1; PPP: phosphoprotein phosphatase; STAT6: signal transducer and activator of transcription 6; OAA: oxaloacetate; α-KG: α-Ketoglutaric acid; FFA: fatty acids; NADH: nicotinamide adenine dinucleotide reduced form; NAD: nicotinamide adenine dinucleotide; ROS: reactive oxygen species.

Tumor-associated immune cells have both inhibitory and promoting effects on progressive tumors, and the balance between them determines the development of tumors [137]. Tumor-associated macrophages (TAMs) are a type of natural immune cell, which are abundant in TME and play a double-edged sword role in tumorigenesis [138]. In the inflammatory tumor microenvironment, the phenotype of TAM changes from M1 (tumor-killing phenotype) to M2 (tumor-promoting phenotype), and changes in cellular energy metabolism are critical for the polarization of TAMs from M1 to M2 [139]. M1-type macrophages maintain survival through high glycolysis and release of lactate under hypoxia, while M2 macrophages maintain energy demand through OXPHOS (Figure 2) [140]. On the one hand, CSCs induce TAMs to produce the M2 phenotype, block the anti-tumor effect of CD8^+^ T cells during chemotherapy and induce M2 macrophages to secrete tumor cell colony-stimulating factor (CSF), transforming growth factor (TGF), VEGF and chemokine ligand [141]. On the other hand, M2-type macrophages can secrete IL-6, IL-10, TGF-β and VEGF, and by activating the STAT3/NF-κB signaling pathway promotes the self-renewal of CSCs, thereby increasing the number, tumorigenicity and drug resistance of CSCs [142]. The activation of these pathways induces the production of cytokines and further recruitment of M2-type macrophages, resulting in a TME that is more conducive to the invasion and migration of CSCs [143]. In particular, IL-10 inhibits cytotoxic T-cell activation by downregulating major histocompatibility complex (MHC) expression on cancer cells and antigen-presenting cells (APCs), and prevents antigen specific T cells from recognizing cancer cells [144].

## 10. Cancer Stem Cell Metabolism and Treatment

Targeting tumor metabolic reprogramming may provide new ideas for tumor prevention and treatment. The significantly enhanced FAO in recurrent LSCs can compensate for the lack of production capacity caused by the imbalance in amino acid metabolism [145]. Acetyl carnitine, which assists the transport of fatty acids into mitochondria, is significantly increased in LSCs with treatment resistance [146]. Inhibition of cellular uptake of fatty acids or inhibition of carnitine acyltransferase activity to block fatty acids from entering mitochondria can restore the sensitivity of LSCs to chemotherapy [146]. FAO plays an important role in maintaining self-renewal and inducing the chemotherapy resistance of BCSCs [128]. Leptin derived from breast adipose tissue can promote the expression of carnitine palmitoyltransferase 1B (CPT1B) through the JAK/STAT3 signaling pathway and thus enhance FAO in BCSCs [147]. In the mouse model of breast cancer, inhibiting the leptin JAK/STAT3-CPT1B axis can restore the chemosensitivity of BCSCs [128]. Recurrent LSCs can increase the level of intracellular NAD^+^ by enhancing nicotine metabolism, and then promote the metabolism of amino acids and fatty acids in cells mediated by NAD^+^, thereby driving and maintaining the OXPHOS of cells [147]. Inhibition of nicotinamide phosphoribosyltransferase, an important rate-limiting enzyme in nicotine metabolism, can significantly reduce the tumor load and the number of LSCs in xenograft models [147]. It is worth noting that, in addition to the change in CSCs’ metabolism directly caused by treatment, TME affects the metabolic phenotype of CSCs through interaction with CSCs, thereby enhancing the therapeutic resistance of CSCs. In chronic myeloid leukemia (CML) animal models, gonadal adipose tissue (GAT) is an important storage pool of LSCs in addition to hematopoietic organs [148]. LSCs in GAT have an obvious pro-inflammatory phenotype, and they can upregulate and secrete a variety of proinflammatory and chemokines, including some of the cytokines TNF-α, IL-1α and IL-1β [148]. CSF2 can promote fat mobilization and decomposition of GAT, and the lipolysis of GAT can in turn enhance the activation of FAO in CD36^+^ LSCs, thus enabling LSCs in GAT to acquire stronger therapeutic resistance than bone marrow LSCs [148]. For advanced gastric cancer, mesenchymal stem cells in tumor tissue secrete a large amount of TGF-β1 that induces the expression of long-chain noncoding RNA MACC1-AS1, thereby enhancing the self-renewal and therapeutic resistance of CSCs mediated by FAO [149]. In addition, the acquired resistance of pancreatic cancer CSCs to gemcitabine has become a huge obstacle in the treatment of advanced pancreatic cancer [150]. It has been found that the hypoxia microenvironment further enhances the chemoresistance of pancreatic cancer CSCs through protein kinase B (AKT)/Notch1-mediated signaling pathway [150].

## 11. Future Perspectives

**Figure 3:**
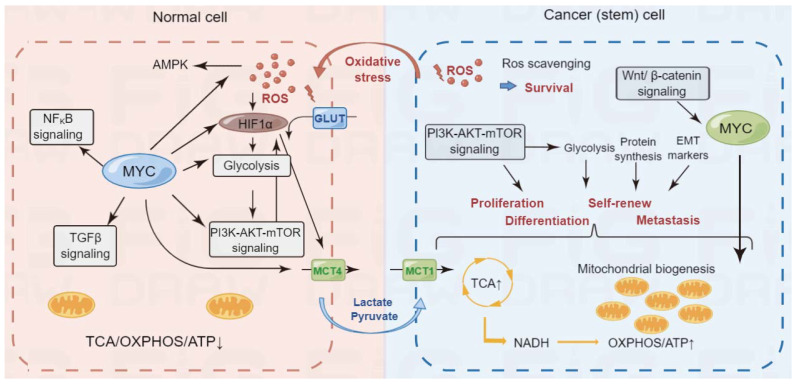
Metabolic difference between normal cells and cancer (stem) cells. Cancer stem cells exhibited enhanced glycolysis, catabolism of glutamate and fatty acids (FAs), which provided more intermediate metabolites tricarboxylic acid (TCA) cycle to generate more ATP. Signaling pathways, such as PI3K-AKT-mTOR pathway, which involved in the metabolic reprogramming in cancer stem cells, were also effective targets for cancer-targeted therapy. OXPHOS: oxidative phosphorylation; NADH: nicotinamide adenine dinucleotide reduced form; NAD: nicotinamide adenine dinucleotide; ROS: reactive oxygen species; EMT: epithelial–mesenchymal transition; GLUT: glucose transporter; MCT1: monocarboxylate transporter 1; MCT4: monocarboxylate transporter 4.

The unique metabolic characteristics of cancer stem cells are one of the reasons for their resistance to treatment and lead to tumor recurrence. Targeting cancer stem-cell metabolism has become a new idea in tumor therapy, but it still faces many problems and challenges. First of all, the metabolic network of CSCs is extremely complex (Figure 3). In this complex network, we still do not fully understand the core genes, but more and more evidence shows that MYC, as a proto-oncogene, regulates the stemness and the metabolism of cancer cells [151,152]. However, it is still uncertain whether MYC can be used as a therapeutic target. Secondly, the effect of the tumor microenvironment on the metabolic reprogramming of CSCs is still unclear. During tumorigenesis, the metabolic characteristics of CSCs will change, and different subsets of CSCs will appear, which increases the difficulty of targeted therapy. In the future, targeted cancer stem-cell metabolic therapy may need to target multiple metabolic pathways at the same time. Although the research on cancer stem-cell metabolism remains to be deepened, targeted cancer stem-cell metabolic therapy strategies have shown great potential and application value in tumor therapy.
